# Explosives Analysis Using Thin-Layer Chromatography–Quantum Cascade Laser Spectroscopy

**DOI:** 10.3390/molecules30081844

**Published:** 2025-04-19

**Authors:** John R. Castro-Suarez, Luis A. Pérez-Almodóvar, Doris M. Laguer-Martínez, José L. Ruiz-Caballero, José A. Centeno-Ortiz, Tamara Felix-Massa, Leonardo C. Pacheco-Londoño, Samuel P. Hernández-Rivera

**Affiliations:** 1 Center for Chemical Sensors and Chemical Imaging and Surface Analysis Center, Department of Chemistry, University of Puerto Rico-Mayagüez, Mayagüez, PR 00681, USA; luis.perez3@upr.edu (L.A.P.-A.); doris.laguer@upr.edu (D.M.L.-M.); jlruiz50@gmail.com (J.L.R.-C.); jacenteno@comcast.net (J.A.C.-O.); tamara.felix@upr.edu (T.F.-M.); 2 Exact Basics Area, Universidad del Sinú, Unisinú, Cartagena 130015, Colombia; 3 Life Science Research Center, Universidad Simón Bolívar, Barranquilla 080002, Colombia; leonardo.pacheco@unisimon.edu.co

**Keywords:** mid-infrared laser spectroscopy, thin-layer chromatography (TLC), trinitrotoluene (TNT), partial least squares regression (PLS), principal components analysis (PCA)

## Abstract

A new hyphenated technique using thin-layer chromatography (TLC) to separate analytes in mixtures, coupled with mid-infrared (MIR) laser spectroscopy for identification and quantification, is presented. The method, which provides a means for rapid screening of analytes that is practical, low-cost, fast, robust, and reproducible, was tested using nitroaromatic and aliphatic nitro high explosives (HEs) as target analytes. HEs are anthropogenic contaminants containing an -NO_2_ group. For validation of the new technique, a direct comparison of the 2,4,6-trinitrotoluene (TNT) spectrum, obtained by attenuated total reflection-Fourier transform infrared (ATR-FTIR) spectroscopy coupled with TLC, was carried out. The MIR laser spectroscopy-based method was evaluated by calculating the analytical figures of merit regarding the calibration curves’ linearity and the method’s sensitivity and precision. The TNT spectrum obtained by the MIR laser method showed two prominent and characteristic bands of the explosive at approximately 1350 cm^−1^ and 1550 cm^−1^ compared to the spectrum acquired by ATR-FTIR. The detection limit calculated for TNT was 84 ng, while the quantification limit was 252 ng. Multivariate analysis was used to evaluate the spectroscopic data to identify sources of variation and determine their relation. Partial least squares (PLS) regression analysis and PLS combined with discriminant analysis (PLS-DA) were used for quantification and classification. The new technique, TLC-QCL, is amenable to a smaller footprint with further developments in MIR laser technology, making it portable for fieldwork.

## 1. Introduction

For the last two decades, the scientific literature on explosives detection has focused on methodologies based on spectroscopic and chromatographic procedures [1,2,3,4,5,6]. A critical aspect of both approaches is that each one, when separately used, can obtain very low limits of detection. The use of chromatographic techniques in field operations has been limited because of the lack of portability of the instrumentation required to implement the various separation techniques and the high cost of coupled detection schemes. Thin-layer chromatography (TLC) provides a streamlined sampling and testing protocol that allows rapid and reproducible separation and identification of a wide range of hazardous materials (drugs, explosives, and their precursors) taken from surfaces and liquids and solids for laboratory and field operation. Spectroscopic techniques have the advantage of being useable in practical on-site conditions, facilitating the quick acquisition of data and leading to prompt decisions based on the information obtained, thus reducing casualties and saving numerous lives. Remarkably, vibrational spectroscopy has been demonstrated as valuable for various applications related to the detection and characterization of threat compounds such as chemical warfare agents and a wide variety of explosives, including homemade explosives and toxic industrial compounds [1,7,8,9,10,11,12,13,14,15,16].

The mid-infrared (MIR) region refers to the spectral window from approximately 350 to 4000 cm^−1^. All molecules have characteristic vibrational signals that can be excited upon interaction with photons from an excitation source within this spectroscopic range, enabling the detection of compounds at trace levels [17]. One of the weaknesses of infrared spectroscopy (IRS) has traditionally been the relatively weak optical power of the excitation source, which leads to absorption, transmission, emission, and reflectance of MIR light. Research into more powerful excitation sources has led to collimated, coherent, and polarized sources. These sources were first developed in 1994 at Bell Laboratories with the invention of quantum cascade lasers (QCLs) [18,19]. QCLs are commercially available, and portable setups allow on-site detection of high-interest chemical and biological threat compounds [20,21,22,23,24].

The primary objective of this work is to couple IRS as a detection/identification technique to TLC to meet the need for reliably identifying separated components. The first in situ FTIR detection of chromatographic spots on a TLC plate was reported in 1975 by Percival and Griffiths [25]. A thin layer (100 μm) of adsorbent on an IR-transparent AgCl support allowed IR transmission measurements of dyes and amino acids at the 1–10 μg level. Thus, the IRS-TLC technique was changed from a qualitative analytical method (TLC used alone) to a confirmatory, quantitative analytical method when TLC was coupled with IRS. When reference spectra are unavailable, valuable information regarding the molecular structure of an analyte may be obtained by spectral interpretation. Coupling broadly tunable MIR lasers with TLC can provide helpful information for identifying unknown materials encountered at a crime scene, including undetonated explosives, enabling personnel to be better informed about the safety precautions needed. QCL-TLC, as a portable hybrid technique for explosives analysis, has not yet been reported. This hybrid method would be most beneficial for two possible uses: (i) pre-blast analysis of bulk materials and (ii) post-blast examination. Overall, portable instrumentation has a two-fold advantage: (i) the speed with which results can be obtained and (ii) avoiding the need to transport potentially dangerous materials to a central laboratory.

Over the last four decades, detection limits have been improved by applying an oil mull to TLC plates to reduce IR scattering [26,27]. In 1978, Fuller and Griffiths [28] demonstrated the viability of diffuse reflectance infrared Fourier transform (DRIFT) spectroscopy to measure methylene blue on silica plates. Since then, several studies have been conducted to explore the potential of TLC-DRIFT analysis [29,30,31,32]. These studies, extensively reviewed by Brown and Beauchemin [33], revealed various conventional TLC stationary phases, such as silica, alumina, and cellulose, and reversed-phase materials used in combination with DRIFT yielded minimum identifiable quantities (identification limits) down to approximately 1 μg. The significant difficulty encountered when using DRIFT as an in situ detection method for TLC is the strong absorption background of the adsorbent, which causes severe interferences within particular spectral regions. For example, silica gel absorbs strongly in the regions from 3100 to 3700 cm^−1^ and from 800 to 1600 cm^−1^, obscuring possible analyte absorption.

In combination with spectroscopic techniques, the use of multivariate analysis (MVA) has become more common. MVA algorithms are typically used to classify data, discriminate analytes from each other, and achieve reliable differentiation with high accuracy [34,35]. MVA can be used to analyze complex data, obtaining accurate results in a short amount of time. When applied to chemical sciences, MVA takes the form of chemometrics. Several algorithms can be used to determine the relations between data sets and find sources of variation. Partial least squares (PLS) [36] or its coupling to discriminant analysis (PLS-DA) are essential processes in chemometrics [24,37,38,39].

A field-deployable detection kit for explosives using thin-layer chromatography (TLC) was developed at Lawrence Livermore National Laboratory (LLNL) [40]. Detection levels in the nanogram range were reported. The kit was later modified to allow for propellant detection [41].

This study developed and tested a technique that allows the detection, identification, and quantification of explosives in complex matrices. This proposed methodology used a mid-infrared (MIR) tunable laser spectrometer to detect separated components. The acquired data were processed with chemometric techniques. The approach quickly allowed rapid and reproducible separation and identification of targeted explosives at relative trace levels (~low ng).

## 2. Results

### 2.1. Separation of TNT and PETN from a Pentolite Formulation

Several organic solvents were used as mobile phases to separate the principal components of a lab-made Pentolite formulation that consisted of a binary mixture of TNT: PETN in a 1:1 ratio. The best mobile phase was a binary mixture of hexane: toluene in a ratio of 1:4. Under these conditions, TNT showed a retention factor of Rf = 0.56 ± 0.01, and that of PETN was Rf = 0.45 ± 0.01. The other solvent systems worked well, but their determined Rf values had lower resolutions. The spot diameters of the samples on the TLC plate were 4 ± 1 mm (PETN) and 6 ± 1 mm (TNT). The average time for the TLC separation of the mixture of HEM was ~10 ± 1 min.

The chromatographic spots corresponding to TNT at various masses (0.39–6.25 µg) were developed with the chromogenic reagent DPA, generating a characteristic orange-brown color. The method is currently used for on-site separation. It should be noted that visual inspection made it impossible to detect masses lower than 1.56 µg. Moreover, the methodology used was invasive and modified the chemical characteristics of the analyte. This justifies the necessity for coupling TLC to a noninvasive technique that can detect chromatographic spots and provide physical information to identify the separated chemical compounds. IRS and Raman scattering can provide vibrational information in fingerprint spectra for the analytes.

### 2.2. Spectral Profiles of TNT

Traditionally, the coupling of TLC and IRS has been approached in two ways. The first approach performs FTIR measurements with an ATR accessory in situ. In other words, the separated compounds are directly analyzed on the TLC plate. Then, spectral interferences are expected from the stationary phase composition due to its intense absorption bands in the MIR region. The second approach is usually more laborious and involves the transfer of the analyte from the TLC plate to an IR-transparent substrate before the FTIR measurements can be made. Over forty years, scientific research has shown that both the in situ and transfer methods can be effective and useful, each having specific advantages and limitations [42].

**Figure 1:**
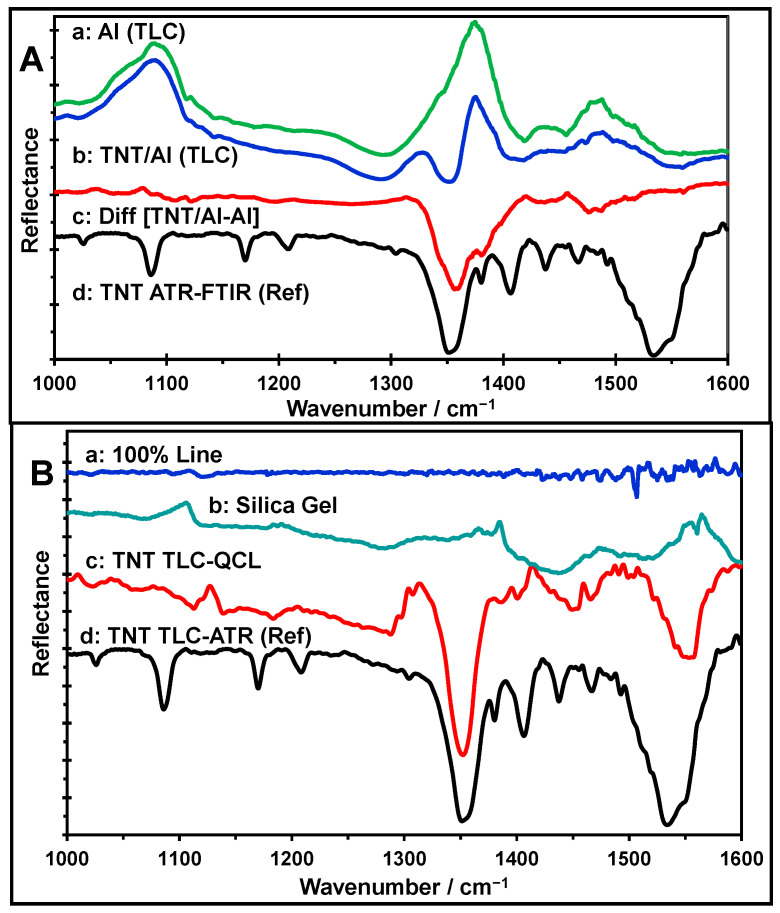
(**A**) (a) Reflectance spectrum using the Al (TLC) substrate as the background; (b) TNT/Al (TLC) with Al (TLC) as the background; (c) difference spectrum of TNT/Al-Al with Al (TLC) as the background; (d) TNT reference spectrum measured by ATR-FTIR. (**B**) (a) 100% line; (b) silica gel spectrum; (c) TNT TLC-QCL spectrum with silica gel as the background; (d) reference TNT spectrum measured by ATR-FTIR.

Spectral measurements were acquired in situ on silica gel-based TLC plates. For the single-beam spectrum of the background (no sample), two alternatives were considered, using aluminum plates (Al) and silica gel (from the TLC plates), as shown in Figure 1. The objective was to determine which of the two background materials provided more straightforward and informative IRS spectra. Figure 1A shows the spectra obtained using Al as the background. The results shown in Figure 1A-a were acquired from the silica gel of the chromatographic plates (labeled TLC). Figure 1A-b shows the spectrum of TNT/TLC. The difference spectrum (TNT/Al-Al) is shown in Figure 1A-c. This procedure was performed to remove the substrate’s IR vibrational signals and obtain the characteristic vibrational signals of TNT. The resulting TNT spectrum shows the distinctive band of the explosive at approximately 1350 cm^−1^. Other MIR bands were not observed using this method compared with the reference spectrum of TNT (Figure 1A-d), which was obtained by ATR-FTIR using a solid TNT sample.

Figure 1B shows the spectra obtained when the silica gel from a TLC plate was used as the background. A single-beam spectrum of a clean area of the silica gel was collected and stored as the background (Figure 1B-b). Immediately afterwards, without moving the TLC plate, another single-beam spectrum was measured and ratioed against the stored background spectrum, resulting in a “100% LINE” spectrum (Figure 1B-a) [43]. This fact illustrated the variation in the noise level across the spectrum. The flatness of the “100% LINE” spectrum indicated that the QCL spectrometer was stable during the sample and reference spectra acquisition periods [43,44]. The single-beam spectrum of the sample was then measured and ratioed against the stored background spectrum to obtain the TNT spectrum on TLC silica gel (Figure 1B-c). This method for recording the MIR spectra of the TLC chromatographic spots of separated analytes is quicker than the methodology previously explained.

Moreover, as Percival and Griffiths [25] have stated, this method allows the interference bands to compensate due to the silica gel and water vapor. The resulting TNT spectrum (Figure 1B-c) shows two intense and representative bands of the HEM at 1350 cm^−1^ and 1551 cm^−1^ and other low-intensity bands compared with the reference spectrum of TNT (Figure 1B-d). This protocol achieved excellent spectral compensation for the silica gel and water vapor bands.

### 2.3. Comparison Between TLC-QCL and TLC-FTIR Methods

**Figure 2:**
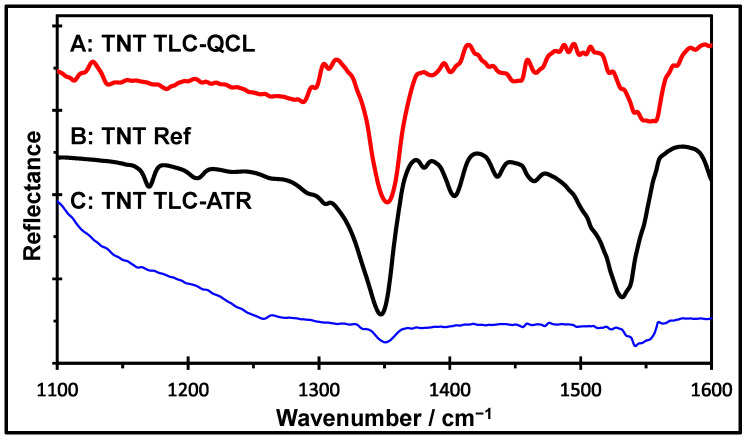
Normalized MIR spectra of TNT and silica gel obtained with various spectroscopic techniques: (A) TNT MIR laser reflectance spectrum (TLC-QCL); (B) TNT reference spectrum obtained by ATR-FTIR; and (C) TNT spectrum obtained using TLC-ATR-FTIR.

To evaluate the capabilities of MIR laser spectroscopy as a technique for the vibrational identification of chemicals on silica gel, TNT was selected as the target analyte. Some of the vibrational bands that were tentatively assigned to TNT were located at 1024 cm^−1^ (corresponding to CH_3_ deformation), 1086 cm^−1^ (assigned to C–H ring in-plane bending), 1350 cm^−1^ (attributed to symmetrical stretching of the nitro groups), and 1551 cm^−1^ (corresponding to asymmetric NO_2_ stretching) [45]. For benchmarking purposes, a comparison was made between the MIR spectra acquired with TLC-ATR-FTIR and with the proposed new technique. Typical TNT spectra obtained by the TLC-QCL method are shown in Figure 2. The TNT spectrum obtained using TLC-ATR-FTIR (Figure 2-C) also shows a high degree of interference from the stationary phase. These results demonstrate that coupling TLC with a tunable QCL can be a viable analytical method. Using TLC-QCL (Figure 2-A), the band’s intensity at 1350 cm^−1^, characteristic of the nitro group vibration, is much higher than that obtained by ATR-FTIR (Figure 2-C). The total integrated band intensity was approximately 154 times higher than the intensity of the band obtained via the FTIR-based method.

### 2.4. Quantification Profiles of TNT on Silicagel TLC Plaes

**Figure 3:**
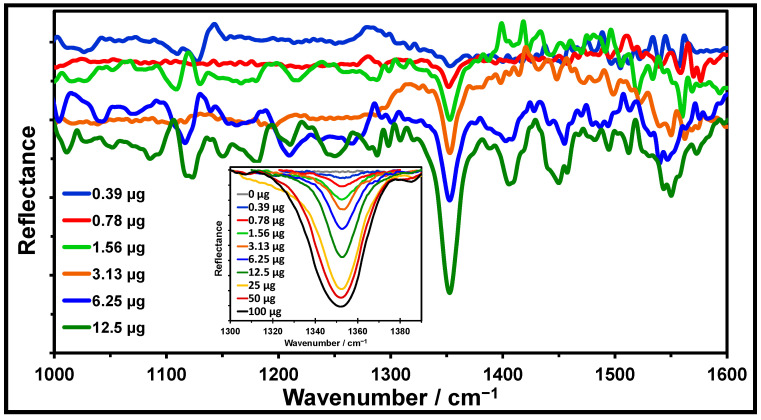
TNT spectra at different concentrations on the TLC silica gel. Baseline correction and smoothing (25 pts) were applied to each spectrum as preprocessing. Inset: details of the NO2 band at 1300–1396 cm^−1^.

Figure 3 shows that the TLC-QCL technique can build robust analytical methodologies for identifying and quantifying selected chemical targets. By depositing TNT onto a silica gel TLC plate at masses ranging from 0.39 to 12.5 µg, the spectral band shapes were very similar to those of the TNT reference spectrum. In Figure 3, as the concentration decreases, the intensities of the MIR vibrational bands decrease according to Beer’s law. At the same time, the IR band at ~1550 cm^−1^ disappears at masses lower than 3.12 µg. This may be due to the rovibrational band of water starting at 1400 cm^−1^ or the strong abortion bands from the silica gel substrate in the region of 1400–1600 cm^−1^. In fact, for low concentrations of TNT deposited onto silica gel plates, an IR band at 1351 cm^−1^ (attributed to the nitro group vibrations) is notably present. Thus, the six spectra shown in Figure 3 demonstrate that the TLC-QCL hybrid technique can be successfully applied to identify explosives.

### 2.5. Quantification by Univariate Analysis

**Figure 4:**
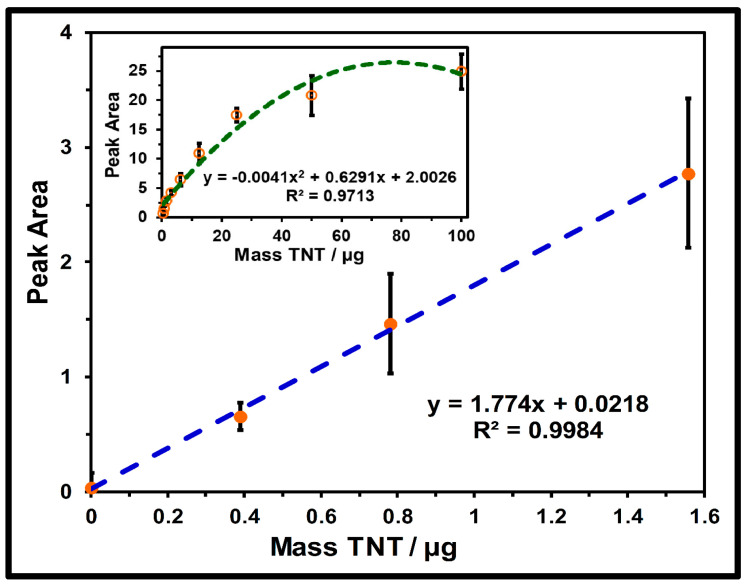
Calibration curve for TNT at different masses deposited from 0.0 to 1.56 µg on the silica gel TLC plates. Inset is the calibration curve at concentrations from 0.0 to 100 µg.

A univariate study was performed on the peak areas as a function of the mass of TNT (µg) deposited onto the silica gel plates to determine the viability of TLC-QCL as a hybrid technique for quantitative analysis. The nitro group symmetric stretching band at 1351 cm^−1^ in the TNT reflectance spectrum, shown in Figure 3, was selected for this study. The inset in Figure 3 shows the baseline-corrected spectrum in 1300–1396 cm^−1^ (OPUS v. 6.0, Bruker Optics, Billerica, MA, USA). A plot of the peak areas vs. mass (µg) of TNT is shown in Figure 4. The response is highly linear for masses up to ~25 µg. A nonlinear dependence was observed over the entire concentration range (inset in Figure 4).

The nonlinearity at high sample concentrations can be attributed to the saturation effects of the sample. According to Beer’s law, a linear response as a function of the analyte concentration holds only for low concentrations, establishing the linear dynamic range of the analytical curve. The high-confidence linear dependence was valid from 0 to 1.56 µg. The analytical FM obtained from the least squares linear regression analysis and the data analyses for several of the values are shown in Table 1. The lowest quantity of explosives that could be detected (DL) for TNT was determined to be 84 ng, and the value for the quantification limit (QL) of TNT was 253 ng using Equations (1) and (2):

DL = (3.3) × (σ/m)(1)

QL = (10) × (σ/m)(2)

where σ is the standard deviation of the intercept and m is the regression line’s slope, both obtained from the calibration curve (Table 1). The smallest sample measured was 0.39 µg under the experimental conditions, resulting in a signal-to-noise ratio (S/N) of 25.8. The band intensities (1336–1366 cm^−1^) were taken as the average height of the six spectra. The noise level (N) was taken as the average root mean square (rms) value of the six spectra measured from 1200 to 1220 cm^−1^, the standard deviation of all data points within this region [46]. The values for the CVs (%) of the spectral response expressed as the peak areas were less than 2%, mostly for the inter-day precision and repeatability of the three concentration levels tested, indicating that the method was precise. The precision values are also included in Table 1.

### 2.6. MVA of the TLC-QCL-Based Methodology

Using the data present in the full MIR laser reflectance spectrum of TNT recorded by TLC-QCL, chemometric analysis methods, such as PLS and PLS-DA, were used to apply full MVA. This procedure allowed for more robust spectral identification, classification, and quantification of our target chemical compound. The PLS Toolbox v. 8.1 for MATLAB (7.10, R2010a) analyzed the spectral data.

For the MVA, the data were divided into two groups: a calibration set and a prediction or test set. The calibration set contained approximately 70% of the data, and the test set comprised the remainder. The test set contained only unseen samples, meaning that spectra from samples used in the calibration set were not included in the test set. PLS-DA was applied to the spectral data to discriminate and classify all the spectra as explosive or no explosive. Some of the main steps taken at this stage were to arrange the spectral data and apply the required preprocessing algorithms to remove or decrease the interference effects from the background and substrate, thereby enhancing the vibrational signature of TNT. PLS-DA was used to decompose the original matrix into two new matrices: one that contained most of the relevant information and the most significant variables in the data and a second that included information less appropriate to the data, generally considered noise.

In PLS-DA, the optimal number of latent variables (LVs) that result in the calibration set’s best classification is determined using cross-validation procedures. The Venetian blind (VB) technique divided the calibration set into 10 cross-validation groups. Preprocessing steps were applied to the data to generate MVA models capable of clustering the spectra based on chemical similarities (i.e., explosive and no explosive). The preprocessing steps used were autoscaling (AS), mean centering (MC), standard normal variate (SNV), first and second derivatives, and their different combinations. Different spectral ranges (windows) between 1000 and 1600 cm^−1^ were analyzed. Of all the MVA models generated, the best ones used the spectral window of 1302.4–1388.4 cm^−1^.

For PLS-DA, classification parameters such as sensitivity (SEN) and specificity (ξ) were used. These were derived from the confusion matrix of the calibration, cross-validation, and prediction sets to evaluate the performance of the classification models. Sensitivity (SEN) is the model’s ability to recognize samples belonging to a class correctly; specificity (ξ) is the ability of the model to reject samples of all other classes. Both SEN and ξ take values between 0 and 1, where 1 is the desired optimal result [37]. Although classification models traditionally focus on accuracy, sensitivity, and specificity, the inclusion of statistical measures (RMSE and R^2^) typically used in quantitative analysis provides a more comprehensive evaluation of the model’s performance. These additional metrics enhance the validity, reproducibility, and applicability of the results, making the methodology more robust and comparable to existing analytical techniques. Table 2 presents a summary of the results obtained from the PLS-DA.

**Figure 5:**
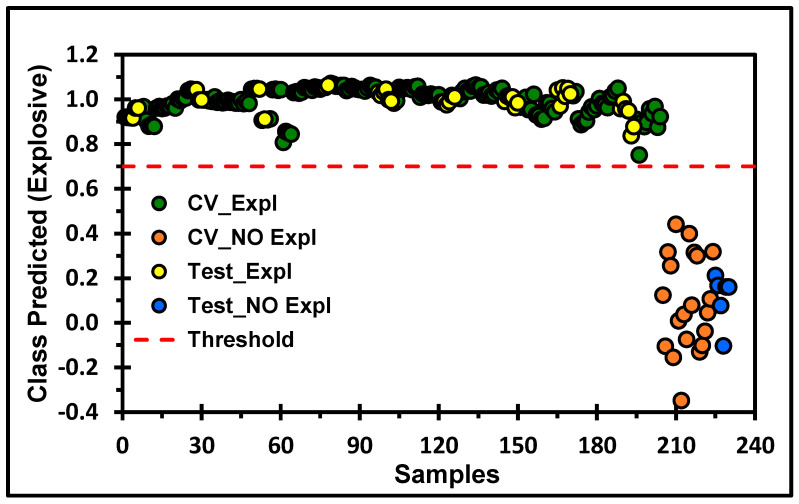
The PLS-DA model for discriminating HEMs on the TLC plate. Class prediction for TNT. The processing steps applied were SNV and MC. The red dotted line represents the threshold for discrimination and a 95% confidence level for clustering.

**Figure 6:**
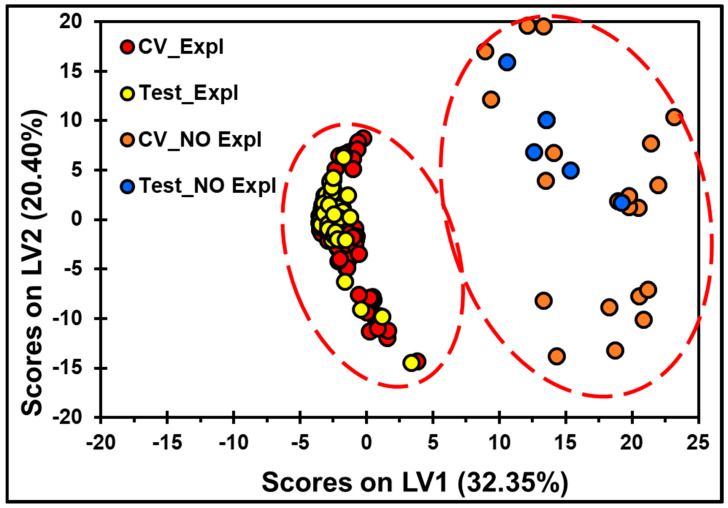
The PLS-DA model for discriminating HEMs on the TLC plates. Score plots of LV2 versus LV1 for the detection of TNT on the TLC plates. The processing steps applied were SNV and MC. The red dotted line represents the threshold for discrimination and a 95% confidence level for clustering.

Figure 5 and Figure 6 show the score plots that resulted from the PLS-DA calculations. The score plots allow visualization of the clustering of the spectral data and show the best results obtained for the generated models after preprocessing was applied. For the MVA, the best models for PLS-DA were obtained by taking the SNV and then applying MC. The class predictions of explosives on the TLC plates from the cross-validation set derived from the PLS-DA models are shown in Figure 5. The discrimination of each spot with the HEM (explosive) on the TLC plate from a clean spot (no explosive) can be observed. Three LVs were required to obtain the best MVA classification model, with SEN and ξ equal to 1 (see Table 2) for TNT from the calibration, cross-validation, and prediction data sets. The variance captured from the matrix was 94.37%, sufficient for good classification of the predicted spectra set for the TLC data. For the MVA of TNT on the TLC plates, six LVs were necessary to capture 80% of the total variance in the spectral data. As shown in the score plots in Figure 6, two LVs, with 52.75% of the spectral variance, were sufficient to obtain excellent classification to distinguish a spot with TNT from a place without TNT. In this model, the spectra from the prediction set (in Figure 6, Test_Expl, and Test_No_Expl) were well grouped with the spectra from the calibration set according to their chemical characteristics.

**Figure 7:**
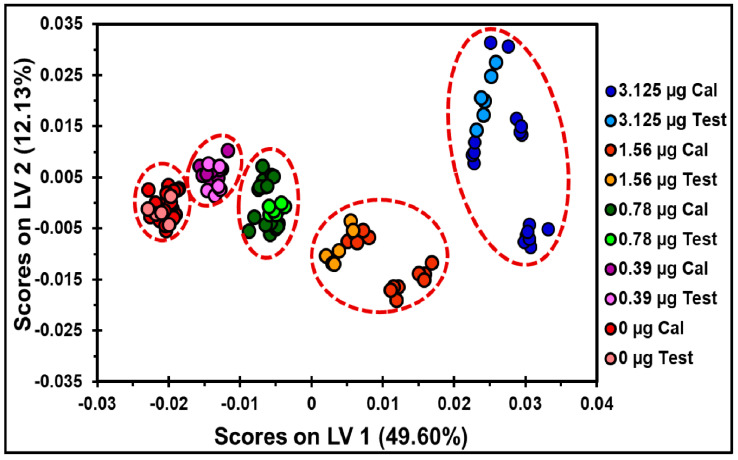
The PLS model for quantifying HEMs on the TLC plates. Score plots of LV2 versus LV1 for the quantity of TNT on the TLC plates. The preprocessing steps applied were the first derivative (15 points) and MC. The red dotted lines represent the threshold for discrimination and a 95% confidence level for clustering. The spectral range used was 1302.4–1388.4 cm^−1^.

In the PLS clustering analysis for the QCL spectra of TNT on the TLC plates, six LVs were required to capture 80% of the total variance in the spectral data using the first derivative (15 points) and MC as the prepossessing steps. Two LVs, corresponding to 61.73% of the variance, were sufficient to obtain excellent spectral classification based on the amount of explosive deposited, between 0 and 3.13 µg, as shown in Figure 7. Using this model, spectra from the prediction set (0–3.13 µg Test) were grouped with spectra from the calibration set according to their vibrational signatures as a function of the amount of TNT deposited.

**Figure 8:**
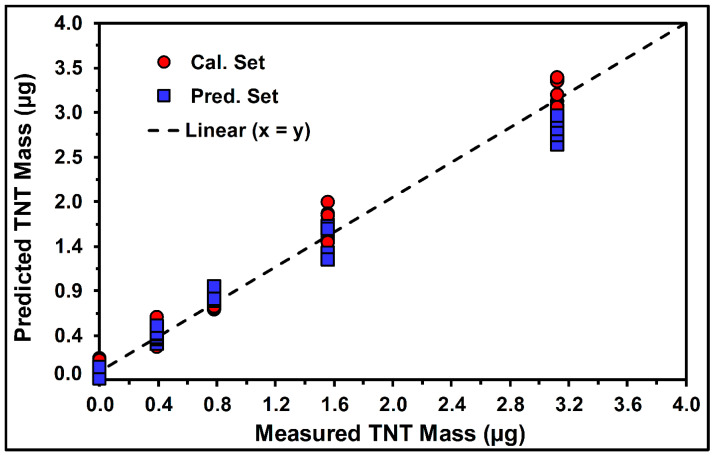
PLS regression plot of the predicted TNT mass vs. measured TNT mass deposited onto the TLC substrates. The preprocessing steps applied were the first derivative (15 points) and MC. The spectral range was 1302.4–1388.4 cm^−1^.

PLS regression was used to analyze the spectral data and find the best correlation between the multivariate spectral information and the TNT mass (µg). Moreover, the root mean square error of cross-validation (RMSECV), root mean square error of prediction (RMSEP), the coefficient of determination from cross-validation (R^2^-CV), and the coefficient of determination from the prediction (R^2^-Pred) were calculated and used as indicators of the quality of the obtained spectral correlations. Chemometric models were obtained based on PLS regression using the measured spectral range (1302.4–1388.4 cm^−1^) and the same sample concentrations as those in the PLS-DA analysis. As a preprocessing step, the first derivative and MC were applied to each spectrum from the dataset. Figure 8 shows a PLS model plot for TNT on the TLC plates. The best results for RMSECV, RMSEP, R^2^-CV, and R^2^-Pred, including the number of LVs required for the PLS model, are shown in Table 3. Three LVs were needed to obtain the best PLS models, resulting in determination coefficients higher than 0.98.

## 3. Materials and Methods

### 3.1. Materials and Reagents

The reagents used in this investigation included high explosives (HEs) such as 2,4,6-trinitrotoluene (TNT), acquired from ChemService (West Chester, PA, USA), and pentaerythritol tetranitrate (PETN; prepared in our laboratory). The solvents used for making the HEM solutions included methanol (99.9%, HPLC grade), dichloromethane (CH_2_Cl_2_, HPLC grade), and acetone (99.5%, GC grade) obtained from Thermo Fisher Scientific (Waltham, MA, USA). The mobile phases used for the chromatographic separations were acetone, hexane, cyclohexane, ethyl acetate, toluene, methanol, petroleum ether, dichloromethane, acetonitrile, 1,2-dichloroethane, and binary mixes of some of these solvents. All solvents were purchased from Sigma-Aldrich (Milwaukee, WI, USA). The aluminum TLC plates, coated with silica gel used for the chromatographic runs, were acquired from Merck (TLC silica gel 60 F254; Merck & Co., Kenilworth, NJ, USA). The chromogenic agent diphenylamine (DPA) (≥99%, ACS grade reagent, Sigma-Aldrich) was employed as a means of visualizing the TNT spots when required.

### 3.2. TLC Protocol

The developing chambers used for the TLC runs were clear glass jars with lids (5 cm diam., 10 cm height), into which the solvents or solvent mixes were transferred (~5 mL) to a depth of just less than 0.5 cm, and for saturation with the solvent vapor, the solvents were allowed to stand. The TLC plates used were cut to 2 cm width × 9 cm height and were carefully handled to avoid damage or contamination of the adsorbent layer. A reference mark was added to the bottom of each plate by drawing a line across the plate at a height of 1.5 cm using a pencil. Ten microliter (10 µL) aliquots of the HEM solutions were added to the plates using a capillary tube, gently touching the plate at the reference mark and allowing the solvent to evaporate. The HEM solutions were prepared by dissolving each sample at a concentration of 625 µg mL^−1^ in acetone, which was subsequently used to generate dilute solutions where the lowest HEM concentration was 39 µg mL^−1^. The prepared TLC plates were placed in the developing chambers and covered. The TLC plates were allowed to develop until the solvent level was approximately 0.5 cm below the top of the plate. Then, the plates were removed from the developing chamber, the solvent front was marked with a pencil, and the plates were allowed to dry. The spots for the separated analytes were detected, marked (with a pencil), and described (shape and size). Colorless spots were detected using a UV lamp. The distances traveled by the solvent and HEMs were measured and the retention times (Rf values) were calculated.

### 3.3. MIR Laser Spectroscopy Instrumentation and Spectra Acquisition

A QCL-based MIR laser pre-dispersive spectrometer (LaserScan^TM^; Block Engineering, Southborough, MA, USA) was used to acquire the back reflection spectra of each detected spot. One of the advantages of this type of source is its high spectral radiance (0.5–10 mW optical power), which is approximately six (6) orders of magnitude brighter than conventional thermal sources (globars). QCL technology has other important features: portability, low energy consumption, long-term power stability, room-temperature operation, and finely tunable output frequency. The MIR spectra were recorded in the spectral range of 990–1600 cm^−1^ at 6 inches from the TLC substrates. In this study, each mass of the explosive per deposited TLC spot was analyzed in triplicate. Each TLC slide was analyzed in two positions (by rotating the TLC slide 90 degrees), and four spectra were acquired (by moving the spectrometer) for each sample spot (three coadds per acquisition) at a spectral resolution of 4 cm^−1^. A total of 24 (3 × 2 × 4) spectra were acquired for each concentration (mass) of the explosive per deposited TLC spot. In addition to a blank solution (with no TNT present), nine TNT standard solutions were prepared in a concentration range that would produce chromatographic spots with masses between 100 and 0.390 µg. Finally, a total of 240 (24 × 10 concentrations) spectra were acquired in this study. The beam spot of the QCL source had an elliptical profile of approximately 2 × 4 mm^2^ at the focal plane. The experiments were performed at room temperature (~21 °C). Spectra using both backgrounds, the TLC silica gel without sample and the aluminum surface (Al), were acquired and compared. To compare the QCL and FTIR spectra, a PerkinElmer Spectrum 100 ATR-FTIR spectrometer was used to acquire the spectra of the spot. The spectra were acquired using 64 scans at a resolution of 4 cm^−1^, with TLC silica gel (without a sample) used as the background.

### 3.4. Univariate Analysis

A univariate method of analysis was used to evaluate the performance of the TLC-QCL-based methodology by calculating the analytical descriptors or figures of merit (FMs) from the results of the experiments. These FMs were defined in terms of the linearity of calibration curves and the method’s sensitivity and precision, according to the International Conference on Harmonization (ICH) [46]. To determine the linearity of the developed method, nine TNT standard solutions were prepared, yielding chromatographic spots with masses ranging from 100 to 0.390 µg, in addition to a blank solution (no TNT present). The chromatographic experiments were evaluated with six different analyses. Linear regression analysis plotted the average peak areas obtained at each concentration. The sensitivity was estimated by calculating the value of the detection limit (DL) and the quantification limit (QL) from the calibration curve using concentrations that showed a linear spectral response. The method’s precision was evaluated in terms of repeatability and intermediate accuracy (inter-day). The reproducibility of the measurements of the peak areas was determined by analyzing three mass levels (high: 100 µg; medium: 25 µg; low: 1.56 µg) four times without changing the position of the TLC plates. The method’s intermediate precision (inter-day) was determined by evaluating six replicas of each of the three specified concentrations over a short period (four days). The peak areas were used to calculate the mean, standard deviation, and coefficients of variation (CVs).

### 3.5. Multivariate Analysis (MVA)

All the spectra were stored in Thermo-Galactic SPC format (Thermo Fisher Scientific, Inc., Madison, WI, USA) and analyzed using PLS and PLS-DA chemometric models generated using the PLS Toolbox, v. 8.1 (Eigenvector Research Inc., Manson, WA, USA) for MATLAB (The MathWorks, Inc., Natick, MA, USA). The tested samples corresponded to the same samples used in the univariate analysis.

## 4. Conclusions

A new hybrid technique is described that couples a quick, easy-to-implement, low-cost separation technique (TLC) with a powerful, laser-based MIR reflectance technique. TLC-QCL can rapidly separate, identify, and quantify analytes of interest, such as HEMs at low concentration levels. The optimal mobile phase for separating the selected target HEM (TNT, R_f_ = 0.56) from the other HEMs was a binary 1:4 solvent mixture composed of hexane and toluene. The spot diameters of the samples were ~0.5 cm, as determined by UV fluorescence. This information was crucial because it allowed locating the sample spots quickly and was very useful for in situ MIR laser sensing. The acquired TNT-QCL spectra exhibited several characteristic bands at 1350–1550 cm^−1^, which compared well with the QCL TNT reference spectrum measured by laser reflectance. In situ spectral measurements using the silica gel TLC plates as the background signal allowed better observation of the MIR vibrational signals of TNT when compared with those obtained using Al plates as the background signal. A DL of 84 ng and a QL of 253 ng were obtained using the calibration curve’s linear range. Comparing the technique with TLC-ATR-FTIR demonstrated that the new hybrid technique was much more sensitive (154-fold) under the same experimental conditions.

Chemometric-based MVA assisted in detecting HEMs deposited onto TLC plates and required only taking the first derivative and applying MC as the preprocessing steps for PLS regression. Robust results were obtained for RMSECV, RMSEP, R^2^-CV, and R^2^-Pred. The preprocessing steps for PLS-DA included SNV and MC. These were sufficient to obtain efficient models with sensitivities and specificities equal to 1.0.

## 5. Patents

Hernandez, S.P.; Castro, J.R. Coupling of Thin-Layer Chromatography (TLC) to Quantum Cascade Laser Spectroscopy (QCLS) for Qualitative and Quantitative Field Analyses of Explosives and Other Pollutants. 2019. U.S. Patent No 10,379,033 B1.

Hernandez-Rivera, et al. Grazing Angle Probe Mount for Quantum Cascade Lasers. 2023. U.S. Patent No 11,567,337 B1.

## Figures and Tables

**Table 1:** Regression analysis of the calibration curve and figures of merit for TNT detection.

Parameters	Value
Linearity range (µg spot^−1^)	0–1.56
Slope (m)	1.774
Standard deviation of slope	0.050
Intercept	0.022
Standard deviation of intercept (σ)	0.045
Regression equation	Peak area:
	1.774 × (Mass TNT) + 0.022
Determination coefficient (R^2^)	0.998
LOD (µg)	0.084
LOQ (µg)	0.253
Precision inter-day (CV, %)	
for 100 µg spot^−1^ (n = 6)	1.090
for 25 µg spot^−1^ (n = 6)	1.369
for 1.56 µg spot^−1^ (n = 6)	3.492
Repeatability (CV, %)	
for 100 µg spot^−1^ (n = 4)	0.809
for 25 µg spot^−1^ (n = 4)	1.005
for 1.56 µg spot^−1^ (n = 4)	2.083

**Table 2:** Summary of the PLS-DA results for the QCL spectra of TNT deposited onto the TLC plate.

Parameters	Data Set	
	Explosive	No Explosive
Sensitivity (Cal)	1.000	1.000
Specificity (Cal)	1.000	1.000
Sensitivity (CV)	1.000	1.000
Specificity (CV)	1.000	1.000
Sensitivity (Pred)	1.000	1.000
Specificity (Pred)	1.000	1.000
Classification error (Cal)	0.000	0.000
Classification error (CV)	0.000	0.000
Classification error (Pred)	0.000	0.000
RMSEC	0.076	0.076
RMSECV	0.096	0.096
RMSEP	0.069	0.069
R^2^ Cal	0.944	0.944
R^2^ CV	0.911	0.911
R^2^ Pred	0.953	0.953
Number of LVs	3	
Variance captured in Y (%)	94.37	

**Table 3:** Statistical parameters of the PLS calibration model for TNT deposited onto the TLC plates.

Parameters	Value
Conc. Range (µg spot^−1^)	0–3.125
Wavenumber Range (cm^−1^)	1302.4–1388.4
Number of LVs	3
Variance Captured (%)	98.63
RMSEC	0.135
RMSECV	0.155
RMSEP	0.210
R^2^ CV	0.982
R^2^ Pred	0.978

## Data Availability

All data supporting the findings of this study are included in the article. The dataset is available on request from the authors.
